# Hydroimplantation versus viscoimplantation: comparison of intraocular lens implantation with and without ophthalmic viscoelastic device in phacoemulsification

**Published:** 2018

**Authors:** Fatih Özcura, Seyit Çevik

**Affiliations:** * Department of Ophthalmology, Kutahya Health Sciences University School of Medicine, Kutahya, Turkey

**Keywords:** balanced salt solution, hydroimplantation, intraocular lens implantation, ophthalmic viscoelastic device, phacoemulsification

## Abstract

**Purpose:** To compare the efficacy and safety of intraocular lens implantation with and without ophthalmic viscoelastic device in phacoemulsification.

**Methods:** A randomized prospective study was conducted on 84 eyes of 84 patients who underwent uneventful phacoemulsification by the same surgeon. Patients were divided into two groups after the completion of lens cortex removal. Intraocular lens implantation was performed with balanced salt solution irrigation in group H (n=42, hydroimplantation) and with ophthalmic viscoelastic device in group V (n=42, viscoimplantation). The main outcomes measured were postoperative changes of intraocular pressure (IOP), central corneal thickness (CCT), mean absolute refractive error, time of surgery, and the frequency of complications. Patients were evaluated 1 day, 1 week, and 1 month postoperatively.

**Results:** There was no significant difference in mean age, gender, preoperative IOP and preoperative CCT between the two groups. IOP and CCT were not significantly different 1 day, 1 week, and 1 month postoperatively between the two groups. Mean absolute refractive error was also not significantly different between the two groups. Time of surgery was significantly lower in group H than in group V (953.81 ± 88.33 seconds, 1072.33 ± 172.16 seconds, respectively, p<0.001). No other complications were observed during the intraocular lens implantation in the two groups.

**Conclusions:** Hydroimplantation technique is safe and effective in phacoemulsification. Furthermore, reduced time of surgery and reduced cost of ophthalmic viscoelastic devices are the advantages of this technique.

**Abbreviations:** OVDs = ophthalmic viscosurgical devices; IOP = intraocular pressure; MARE = mean absolute refractive error; CCT = central corneal thickness; CDE = cumulative dissipated energy; AL = axial length; ACD = anterior chamber depth; ACM = anterior chamber maintainer; ECCE = extracapsular cataract extraction

## Introduction

The introduction of Healon (sodium hyaluronate 1%; Advanced Medical Optics, Inc.) in 1980 for use in intraocular surgery represented a major advance and synergistically accelerated the acceptance of phacoemulsification [**1**]. Cataract surgeons can choose from various ophthalmic viscosurgical devices (OVDs) that have been made commercially available on the market since the introduction of Healon. Their basic function is to create anterior chamber depth, protects the corneal endothelium and the posterior capsule. It also allowed surgeons to stabilize and maintain workspace, protect intraocular tissues, perform capsulorhexis, and facilitate intraocular lens implantation [**1**,**2**].

However, residual OVDs in the anterior chamber can cause some postoperative complications such as intraocular pressure (IOP) spikes, capsular block syndrome, and toxic anterior segment syndrome [**2**,**3**]. Therefore, the OVDs must be removed completely at the end of the surgery in order to avoid these complications. It is a more rational approach to use OVDs in a limited manner to reduce these disadvantages. In this context, some cataract surgeons have preferred the hydroimplantation technique of intraocular lens implantation without OVDs. Tak [**4**] was the first to describe the hydroimplantation technique. In this technique, intraocular lens implantation was performed under continuous balanced salt solution irrigation without OVDs. Other limited studies in literature reported safety of hydroimplantation technique [**5**-**7**]. The aim of this study was to compare the efficacy and safety of intraocular lens implantation with and without ophthalmic viscoelastic device, and to compare postoperative mean absolute refractive error (MARE) not studied earlier.

## Methods

This randomized, prospective study was conducted in the Department of Ophthalmology, Kutahya Health Sciences University School of Medicine. The study was performed in accordance with the Declaration of Helsinki principles and the local Medical Ethics Committee approved the study. An informed consent was obtained from all participants before the study.

## Subjects

Eighty-four eyes of 84 subjects who underwent uneventful phacoemulsification were enrolled in the study. All the study participants underwent detailed ophthalmologic examination including best-corrected visual acuity, measurements of IOP, slit-lamp biomicroscopy, and fundoscopy. Degree of lens opacification was graded by means of a Lens Opacities Classification System (LOCS III). Measurements of central corneal thickness (CCT) were performed before and after the surgery. Patients having complicated cataract, previous ocular surgery or trauma, small pupil, narrow anterior chamber, high myopia and coexisting ocular disorders (corneal disorder, glaucoma, pseudoexfoliation, uveitis, and retinal disorder) were excluded from the study.

## Surgical Technique

A single experienced surgeon (F.O.) performed all of the operations using a peristaltic phacoemulsification machine (Centurion Vision System, Alcon Laboratories Inc., Fort Worth, TX, USA). After anesthesia with 0.5% topical proparacaine solution (Alcaine, Alcon Laboratories Inc., Fort Worth, TX, USA), a 2.2 mm clear corneal incision was made on the steeper corneal meridian. Sodium hyaluronate 3.0% was filled into the anterior chamber and then continuous curvilinear capsulorhexis was performed. Hydrodissection and hydrodelineation were performed to achieve free rotation of the nucleus. Nucleus was emulsified with crater and split technique, the cortex was removed using irrigation and aspiration. Cumulative dissipated energy (CDE) value of the subjects was noted. CDE for phacoemulsification was calculated as CDE = average U/S power × U/S time.

Patients were divided into two groups after the completion of lens cortex removal. One-piece spherical acrylic intraocular lens (Sensar AAB00, AMO) implantation was performed with balanced salt solution (BSS) irrigation in group H (n=42, hydroimplantation) and with sodium hyaluronate 1.4% in group V (n=42, viscoimplantation). The incision was self-sealing and mild edema around the incision site was induced by hydration.

## Patient Evaluation

Patients were evaluated 1 day, 1 week, and 1 month postoperatively. All patients underwent routine ophthalmic examination at every follow-up visit but only measurements of IOP were performed at six hours after the surgery. IOP measurements were performed with the non-contact tonometer (CT-1P Topcon Corporation, Tokyo, Japan). Contact tonometers such as Goldmann applanation tonometer did not use the IOP measurements because of the possible risk of any contamination. CCT was measured with non-contact partial coherence interferometry optical biometers (AL-Scan, Nidek Co, Ltd., Gamagori, Japan) before and after the surgery. MARE was defined as the difference between attempted predicted target refraction and the achieved postoperative spherical equivalent refraction.

## Statistical Analysis

Statistical analyses were performed using the Statistical Package for Social Sciences (version 17.0, SPSS Inc., Chicago, IL, USA). The normality of the continuous variables was evaluated with the Shapiro-Wilk test. The independent samples t test was used for the comparison of the groups. P-values of less than 0.05 were considered statistically significant.

## Results

Demographic data of the all subjects are shown in **Table 1**. There were no significant differences between group H and group V with regard to age and gender.

**Table 1:** Demographic data of the subjects

	Group H	Group V	P
n	42	42	
Age (year)			
Mean±SD	64.36±9.37	65.88±10.72	0.490
Range	38 – 86	42 – 88	
Gender			
Male	29	29	1
Female	13	13	
H = hydroimplantation, V = viscoimplantation, SD = standard deviation			

Mean IOP, CCT, axial length (AL), anterior chamber depth (ACD), keratometry, LOCS III, and CDE values in control and study eyes are highlighted in **Table 2**. There was no significant difference in those variables between the two groups.

**Table 2:** Mean IOP, CCT, AL, ACD, keratometry, LOCS, and CDE values of subjects

	Group H	Group V	P
n	42	42	
IOP (mmHg)			
Mean±SD	16.95±3.23	17.29±2.87	0.618
Range	10 – 25	12 – 23	
CCT (µm)			
Mean±SD	542.36±32.01	538.29±28.15	0.541
Range	463 – 604	480 – 586	
AL (mm)			
Mean±SD	23.58±0.88	23.54±0.75	0.823
Range	21.94 – 26.11	22.12 – 24.99	
ACD (mm)			
Mean±SD	3.28±0.37	3.30±0.35	0.892
Range	2.39 – 3.93	2.44 – 4.01	
Keratometry (D)			
Mean±SD	43.54±1.61	43.37±1.68	0.632
Range	39.00 – 49.00	40.00 – 47.00	
LOCS III			
Mean±SD	3.98±1.20	3.83±1.32	0.606
Range	2 – 5	2 – 5	
CDE			
Mean±SD	4.42±3.46	4.18±2.83	0.727
Range	0.27 – 14.86	0.38 – 11.28	
H = hydroimplantation, V = viscoimplantation, SD = standard deviation, IOP = intraocular pressure, CCT = central corneal thickness, AL = axial length, ACD = anterior chamber depth, D = diopter, LOCS = lens opacities classification system, CDE = cumulative dissipated energy			

**Fig. 1:**
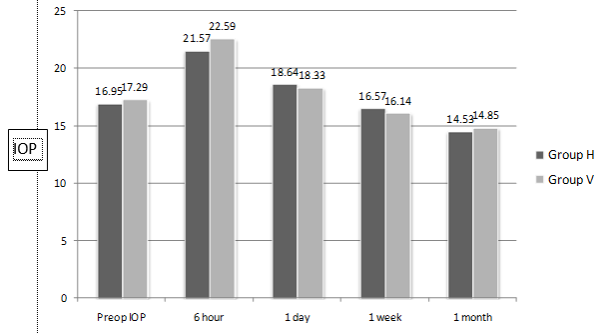
Postoperative course of IOP

**Fig. 2:**
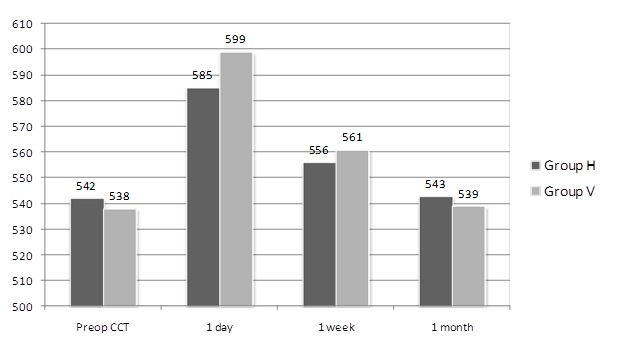
Postoperative course of CCT

**Fig. 1** and **2** show postoperative course of IOP and CCT, respectively. IOP and CCT were not significantly different 1 day, 1 week, and 1 month postoperatively between the two groups.

MARE was lower in group H than in group V but this difference was not significant between the groups (0.31 ± 0.21 D, 0.40 ± 0.23 D, respectively, p=0.099). Time of surgery was significantly lower in group H than in group V (953.81 ± 88.33 seconds, 1072.33 ± 172.16 seconds, respectively, p<0.001). No other complications were observed during the intraocular lens implantation in the two groups.

## Discussion

Various postoperative complications, especially IOP spikes, can arise when remnant OVDs are noticed after the surgery [**2**,**3**]. No significant postoperative IOP or CCT difference was determined between the hydroimplantation and viscoimplantation groups in this study. Also we did not encounter any complications during the intraocular lens implantation with hydroimplantation technique.

Tak [**4**] first used the term “hydroimplantation” in literature, but intraocular lens implantation has been reported under the continuous balanced salt solution irrigation without OVDs earlier. Continuous balanced salt solution irrigation by anterior chamber maintainer (ACM) system is the standard technique for the extracapsular cataract extraction (ECCE) surgery before the advent of OVDs. Balanced salt solution had the disadvantage of easily escaping from the large corneal incision, resulting in anterior chamber collapse, iris prolapse, and corneal endothelium injury. The use of OVDs was rapidly common because of significant benefit for corneal endothelium in large corneal incision period [**5**,**6**,**8**].

Wright et al. assessed the safety and efficacy of ACM without the OVDs in Mini-Nuc ECCE technique on 46 patients. They reported that small incision cataract surgery using the ACM in terms of visual outcome and induced astigmatism is comparable with the results obtained using other techniques that utilize a similar size of incision. However, in view of the magnitude and range of the endothelial cell losses associated with this technique, the concurrent use of viscoelastic is suggested [**9**]. Shingleton and Mitrev evaluated the safety and efficacy of an ACM versus a viscoelastic material for IOL implantation on 33 patients who had consecutive bilateral cataract extraction by phacoemulsification. They reported no serious intraoperative or postoperative complication and the use of ACM for IOL implantation had lower IOP on the first postoperative day [**10**].

As Tak reported earlier [**8**], according to the ACM system for IOL implantation the hydroimplantation technique has some advantages and we have also experienced the following: because no extra port was required, eye stabilization and centration was better, and the tip of the irrigation cannula could be used for other maneuvers when required; e.g., unfolding the leading haptic, preventing tilting of the optic, protecting the corneal endothelium by keeping the irrigation cannula between the IOL dialer and the endothelium, and with the IOL dialer to bimanually rotate the IOL optic to the desired final position. Limited studies reported the usefulness of the hydroimplantation technique. Studeny et al. reported no significant differences in terms of endothelial cell loss, postoperative changes of IOP, and the frequency of complications between the hydroimplantation and standard viscoimplantation [**5**]. Ogurel et al. evaluated the safety of hydroimplantation in cataract surgery in patients with pseudoexfoliation syndrome. They reported that CCT, IOP, and corneal endothelial cell count were not different between the hydroimplantation and viscoimplantation groups, but IOP was lower in the hydroimplantation group at 24h after surgery [**6**].

Postoperative refractive error is very important after the cataract surgery. The aim of the cataract surgery is not only to restore clear media and vision but also to achieve the predicted postoperative refractive status. MARE was not studied in hydroimplantation technique earlier. More predictable refractive results were found in hydroimplantation technique but the difference was not statistically significant. Ozates et al. reported that the hydroimplantation technique induces central placement of one-piece foldable acrylic intraocular lenses postoperatively by reducing decentration and the angle of tilt [**7**]. This report is supported by the low postoperative refractive error after the phacoemulsification with hydroimplantation technique. Another advantage of the hydroimplantation technique is that it significantly reduces the duration of the surgery. Reduced time of surgery was found at approximately one hour and a half in our study. Ogurel et al. reported that reduced time is approximately three minutes [**6**]. A recent study by Chen et al. compared the hydroimplantation technique with the conventional implantation on toric intraocular lens and concluded that the hydroimplantation technique provided advantages of increased efficiency, reduced surgical time and cost, and no concerns of OVD-induced elevated IOP [**11**].

As a conclusion, the hydroimplantation technique is safe, useful, and effective in phacoemulsification. Furthermore, reduced time of surgery, lower postoperative refractive error, and reduced cost of OVDs are the advantages of this technique.

**Conflict of interest**

The authors do not have any financial or proprietary interest in a product, method, or material mentioned in the manuscript.
